# CircSETD3 (hsa_circ_0000567) inhibits proliferation and induces apoptosis in cholangiocarcinoma cells via downregulation of microRNA-421 expression

**DOI:** 10.1080/21655979.2022.2061283

**Published:** 2022-04-17

**Authors:** Wei Xiong, Aiqing Zhang, Xiuli Xiao, Wenjuan Liu

**Affiliations:** a Digestive Endoscopy Center, Mengchao Hepatobiliary Hospitai of Fujian Medical University, Fuzhou, Fujian, China; b Department of Gastroenterology, Shanxi Bethune Hospital, Shanxi Academy of Medical Sciences, Tongji Shanxi Hospital, Third Hospital of Shanxi Medical University, Taiyuan, Xiaodian. District China

**Keywords:** CircSETD3, miR-421, cholangiocarcinoma cells, BMF, BAX, Bcl-2

## Abstract

Cholangiocarcinoma (CCA) is a fatal tumor associated with chronic inflammation. Circular RNAs (circRNAs) have been evidenced to be involved in tumorigenesis and tumor progression. This study aimed to explore the effects and potential molecular mechanism of circSETD3 in CCA progression. Levels of CircSETD3 and microRNA (miR)-421 in CCA tissue and cell lines were measured using quantitative real-time polymerase-chain reaction (qRT-PCR). A direct target of miR-421 was predicted using TargetScan and further confirmed by a dual-luciferase reporter assay. Cell proliferation and apoptosis were measured using MTT (3-(4,5-dimethylthiazol-2-yl)-2,5-diphenyltetrazolium bromide) assay and flow cytometry, respectively. The activity of caspase-3 was also examined using caspase-3 activity detection kits. Moreover, the levels of B-cell lymphoma-2 modifying factor (*BMF*), B-cell lymphoma 2 (*BCL2*), and Bcl-2-associated X protein (*BAX*) in TFK1 cells were assessed using qRT-PCR and western blot analysis. We found that circSETD3 was downregulated, while miR-421 was upregulated in CCA tissues and cell lines. CircSETD3 negatively regulated miR-421 levels in TFK1 cells. Functional assays revealed that circSETD3-plasmid inhibited cell proliferation, induced apoptosis, promoted caspase-3 activity, enhanced Bax and cleaved-Caspase 3 expression, and reduced Bcl-2 levels, and these effects were reversed by miR-421 mimic. Meanwhile, similar results were observed in miR-421 inhibitor-transfected TFK1 cells, and these results were abolished by BMF-siRNA. BMF, a direct target of miR-421, was downregulated in CCA tissues and cell lines. These findings demonstrate that circSETD3 inhibits proliferation and induces apoptosis in CCA cells by regulating the miR-421/BMF axis, indicating its potential as a promising candidate for CCA therapy.

## Graphical abstract

**Figure:**
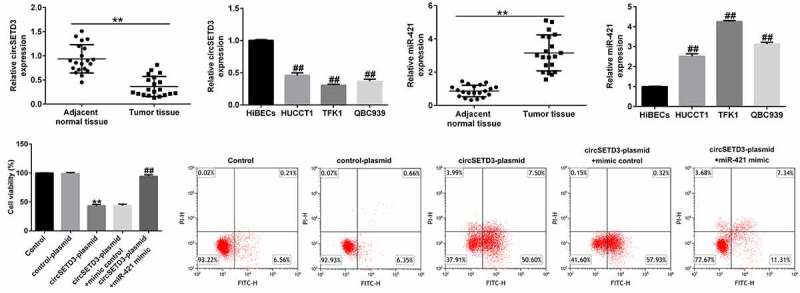

## Research highlights

- CircSETD3 is downregulated and miR-421 was upregulated in CCA cells and tumor tissues;
- CircSETD3-plasmid inhibits TFK1 cell viability and promotes apoptosis by regulating miR-421;
- miR-421 inhibitor suppresses proliferation and induces apoptosis in TFK1 cells through BMF.

## Introduction

Cholangiocarcinoma (CCA) is a malignant tumor originating from the extrahepatic bile duct, including the bile duct from the hilar region to the lower end of the common bile duct [1,2]. Most patients with CCA have jaundice, weight loss, abdominal pain, sometimes accompanied by fever, abdominal mass, and other symptoms [3]. In recent decades, the annual incidence rate of CCA worldwide has been increasing. Surgical treatment, radiotherapy, and chemotherapy are used for CCA treatment in the clinical setting [4]; however, most patients with CCA are diagnosed as advanced, and the five-year survival rate ranges from approximately 20% to 40% [5]. In recent years, new approaches such as gene therapy hold promise for significantly improving survival for cancer patients [6]. Therefore, comprehensive study of the pathogenesis of CCA and identification of new therapeutic targets are key to improving the treatment of CCA.

Circular RNA (circRNA), a special kind of noncoding RNA molecule, has a closed circular structure and is not affected by RNA exonuclease. It was reported that circRNAs are rich in microRNA (miRNA) binding sites and play the role of miRNA sponge in cells [7]. Studies have shown that circRNAs have important effects on the growth, proliferation, apoptosis, invasion, and metastasis of malignant tumor cells [8,9]. Tang et al. revealed that circSETD3 regulates MAPRE1 through miR-615-5p and miR-1538 sponges to promote migration and invasion in nasopharyngeal carcinoma [10]. Moreover, a report by Xu et al. suggested that circSETD3 (hsa_circ_0000567) acts as a sponge for miR-421, inhibiting hepatocellular carcinoma growth [11]. However, the role of circSETD3 in CCA remains unclear.

miRNAs are a class of noncoding RNAs that are 19–25 nucleotides long. Some miRNAs also have tissue specificity, mainly by targeting the 3’-untranslated region (UTR) of mRNAs, and play a role in RNA silencing and post-transcriptional regulation of multiple target genes [12,13]. Abnormal expression of miRNAs has been detected in various types of malignant tumors. For example, miR-421 is significantly upregulated in human gastric cancer and promotes cell proliferation [14]. In addition, miR-421 inhibited the proliferation and metastasis of colorectal cancer by targeting MTA1 [15]. Besides, miR-421 has been revealed to function as an oncogenic miRNA in biliary tract cancer [16]. However, whether miR-421 can regulate the biological functions of CCA cells and its latent mechanism require further exploration.

In the present study, we hypothesized that circSETD3 affects CCA cell proliferation and apoptosis through the regulation of miR-421. Therefore, this study aimed to investigate the possible roles of circSETD3 and miR-421 in CCA and elucidate the underlying mechanisms. Our results proved that circSETD3 inhibits proliferation and induces apoptosis in CCA cells by regulating the miR-421/BMF axis. Our findings identified circSETD3 as a novel therapeutic target for CCA, providing a theoretical basis for CCA treatment.

## Materials and methods

### Clinical specimen collection

A total of 20 CCA and adjacent normal tissues were collected from 20 CCA patients (16 perihilar cholangiocarcinoma patients; 4 intrahepatic cholangiocarcinoma patients) who underwent surgical treatment at the Mengchao Hepatobiliary Hospital of Fujian Medical University. All specimens were rapidly frozen, stored in liquid nitrogen, and preserved at −80°C for further analysis. The experimental procedures were approved by the Ethics Committee of Mengchao Hepatobiliary Hospital of Fujian Medical University. Written informed consent and permission to use the tissues were obtained from all the patients.

### Cell culture

Human intrahepatic bile duct epithelial cells (HiBECs) and CCA cell lines (HUCCT1, TFK1, and QBC939) were obtained from American Type Culture Collection (USA). The cells were cultured in Roswell Park Memorial Institute-1640 medium (Gibco, USA) containing 15% fetal bovine serum (Gibco) and 1% penicillin/streptomycin (Gibco) in a humidified incubator containing 5% CO_2_ at 37°C.

### Quantitative reverse transcription polymerase chain reaction (qRT-PCR) analysis

After treatment, the levels of circSETD3, miR-421, *BCL2*, and *BAX*, were measured by qRT-PCR. RNA was isolated from HUCCT1, TFK1, QBC939 and HiBECs using the RNA-isolation kit (Life Technologies, USA) following the manufacturer’s protocol. Then, the total RNA was reverse transcribed to cDNA using PrimeScript RT Reagent Kit (TaKaRa Bio, Inc., China) following the manufacturer’s protocol, and qRT-PCR analysis was performed using the SYBR PrimeScript RT-PCR Kit (TaKaRa) with ABI 7500 Real-Time PCR System (Agilent Technologies, USA). The relative gene expressions were calculated using the 2^−ΔΔCt^ method [17].

### Cell transfection

CircSETD3-plasmid, control-plasmid, miR-421 mimic, mimic control, inhibitor control, miR-421 inhibitor, BMF-siRNA, or control-siRNA was transfected into TFK1 cells using Lipofectamine 2000 (Life Technologies Corporation, USA) for 48 h according to the manufacturer’s instructions. qRT-PCR was performed to evaluate cell transfection efficiency.

### Dual-luciferase reporter assay

TargetScan was used to predict the potential targets of miR-421 [18]. The 3’-UTR of *BMF* containing miR-421 binding sites was cloned into pMIR vectors (Ambion, USA) to generate the BMF wild-type (BMF-WT) or BMF mutated (BMF-MUT) plasmid. For the reporter activity analysis, 293 T cells were cotransfected with BMF-WT or BMF-MUT plasmids and miR-421 mimic or mimic control using Lipofectamine 2000 (Invitrogen, USA) according to the manufacturer’s protocol. After 24 h, luciferase activity was measured using the Dual-Luciferase Reporter Assay System (Promega, USA) [19].

### MTT (3-[4,5-dimethylthiazol-2-yl]-2,5-diphenyl tetrazolium bromide) assay

Cell viability was analyzed using MTT assay [20]. After treatment, TFK1 cells were seeded into 96-well plates, treated with 10 μL MTT solution, and continuously incubated for 4 h. Then, the supernatant was discarded and 100 μL of dimethylsulfoxide was added to dissolve the blue formazan crystals. Finally, the optical density was measured at a wavelength of 490 nm using a microplate reader (Jupiter G19060; Dorval, Canada) following the manufacturer’s instructions.

### Western blot analysis

After treatment, the total proteins were extracted from TFK1 cells with radioimmunoprecipitation assay lysis buffer (Beyotime, China) and measured using BCA Protein Assay Kit (Invitrogen) following the manufacturer’s protocol. Then, proteins were separated in 10% sodium dodecyl sulfate–polyacrylamide gel electrophoresis and transferred onto a polyvinylidene fluoride membrane. After blocking with 5% skim milk in phosphate-buffered saline with Tween 20 for 1 h, the membranes were incubated with primary antibodies against GAPDH, Bcl-2, Bax, cleaved-Caspase 3, and BMF (1:1000 dilution) overnight at 4°C. The membranes were then washed and incubated with a secondary antibody at room temperature for 1 h. Finally, the protein bands were visualized using enhanced chemiluminescence detection system reagents (Pierce, Rockford, USA) and quantified using ImageJ Software [21].

### Flow cytometry analysis

After treatment, TFK1 cells were detected by a double staining apoptosis detection kit (Beyotime) [22]. Annexin V-fluorescein isothiocyanate and propidium iodide were added to the TFK1 cell suspension for 30 min at 37°C in the dark following the manufacturer’s instructions. Finally, apoptotic cells were checked and analyzed using a flow cytometer (BD Biosciences, USA).

### Detection of caspase-3 activity

After treatment, Caspase-3 Activity Assay Kit (Beyotime) was used to detect caspase-3 activity in TFK1 cells following the manufacturer’s instructions [23]. Briefly, the cells were dissolved with a lysis buffer, and the supernatant of the lysate was collected and centrifuged for 10 min. Then, the supernatant was cultured in Ac-DEVE-pNA and reaction buffer at 37°C for 2 h. A microplate reader (BioTek, USA) was applied to detect the optical density at 405 nm.

### Statistical analysis

Statistical analysis was performed with GraphPad Prism (version 6.0; GraphPad Software, San Diego, CA, USA). All results are expressed as the mean ± standard deviation from three independent experiments. Differences between multiple groups were analyzed using a one-way analysis of variance and Student’s *t*-test was used for the comparison between the two groups. Statistical significance was set at *P*< 0.05.

## Results

### CircSETD3 is downregulated and miR-421 was upregulated in CCA cells and tumor tissues

**Figure 1.**
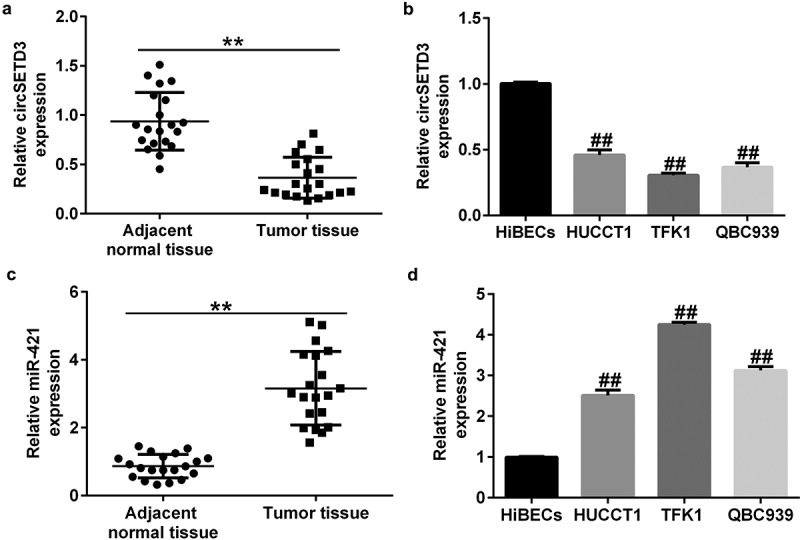
Expression of circSETD3 and miR-421 in CCA tissues and cell lines.

Previous studies have identified miR-421 as an oncogene in biliary duct carcinoma. Moreover, circSETD3 (Hsa_circ_0000567) acts as a sponge of miR-421 to inhibit the growth of hepatocellular carcinoma. We first evaluated the levels of circSETD3 and miR-421 in 20 CCA and 20 normal tissues by qRT-PCR analysis. Our data revealed that the level of circSETD3 was much lower in CCA tissues than in paracarcinoma tissues (Figure 1(a)). Meanwhile, we observed a downregulation of circSETD3 in CCA cell lines (HUCCT1, TFK1, and QBC939) in comparison with HiBECs (Figure 1(b)). Besides, miR-421 was remarkably upregulated in CCA tissues compared with paracarcinoma tissues (Figure 1(c)). Additionally, we observed an upregulation of miR-421 in CCA cell lines (HUCCT1, TFK1, and QBC939) in comparison with HiBECs (Figure 1(d)). Since the expression of CircSETD3 and miR-421 in TFK1 cells was more distinct from that in HiBECs, we selected the TFK1 cell line for subsequent experiments.

qRT-PCR analysis of circSETD3 (Aa) and miR-421 (c) expression in CCA and adjacent normal tissues. Relative expression levels of circSETD3 (b) and miR-421 (d) in CCA cell lines (HUCCT1, TFK1, and QBC939) and HiBECs were determined using qRT-PCR.

### CircSETD3 negatively regulates miR-421 expression in TFK1 cells

**Figure 2.**
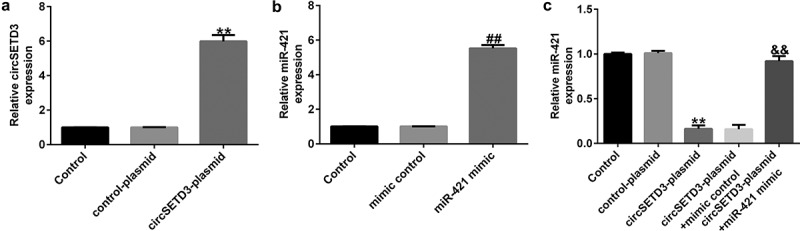
CircSETD3-plasmid regulates miR-421 expression in CCA cells.

To determine the functions of CircSETD3 and miR-421 in CCA cells, circSETD3-plasmid, control-plasmid, miR-421 mimic, or mimic control were transfected into TFK1 cells for 24 h. As shown in Figure 2(a), the circSETD3-plasmid significantly promoted circSETD3 expression in TFK1 cells compared with the control-plasmid. Moreover, miR-421 was evidently downregulated in miR-421 mimic-transfected cells compared with mimic control-transfected cells (Figure 2(b)). We also observed that circSETD3-plasmid remarkably suppressed miR-421 expression in TFK1 cells, and this decrease was reversed by miR-421 mimic (Figure 2(c)). These findings verify that circSETD3 negatively regulates miR-421 expression in TFK1 cells.

CircSETD3-plasmid, control-plasmid, miR-421 mimic, or mimic control were transfected into TFK1 cells for 24 h. (A) qRT-PCR analysis of circSETD3 in circSETD3-plasmid- or control-plasmid-transfected TFK1 cells. mRNA levels of miR-421 in TFK1 cells (B) and circSETD3-plasmid- or circSETD3-plasmid+miR-421 mimic-transfected TFK1 cells (C) were evaluated using qRT-PCR.

### CircSETD3-plasmid inhibits TFK1 cell viability and promotes apoptosis by regulating miR-421

**Figure 3.**
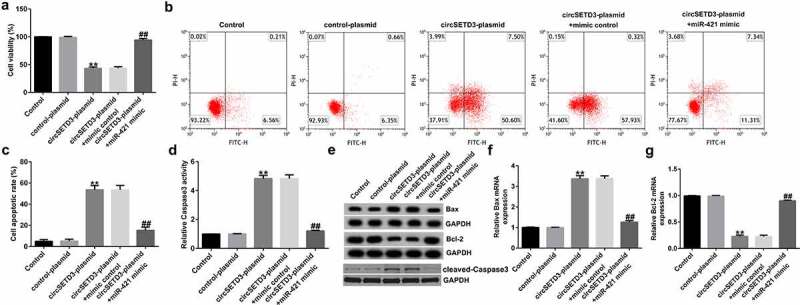
CircSETD3 regulates CCA cell viability and apoptosis by targeting miR-421.

To understand the biological behavior of TFK1 cells coregulated by circSETD3 and miR-421, TFK1 cells were transfected with circSETD3-plasmid, control-plasmid, circSETD3-plasmid+miR-421 mimic, or circSETD3-plasmid+mimic control for 24 h. Then, MTT and flow cytometry assays were conducted to evaluate the effect of circSETD3-plasmid or miR-421 mimic on cell apoptosis and proliferation. The results demonstrated that the circSETD3-plasmid significantly inhibited cell proliferation (Figure 3(a)) and induced more apoptotic cells than the control-plasmid (Figure 3(b -c)). In addition, circSETD3-plasmid promoted caspase-3 activity (Figure 3(d)), increased *BAX* and cleaved-Caspase3 expression (Figure 3(e-f)), and suppressed *BCL2* levels in TFK1 cells (Figure 3(e-g)) compared with control-plasmid. However, all these findings were reversed by miR-421 mimic, indicating that miR-421 mimic reversed the antiproliferation effect of circSETD3-plasmid in TFK1 cells.

TFK1 cells were transfected with circSETD3-plasmid, control-plasmid, miR-421 mimic, or mimic control for 24 h. (a) Cell proliferation was evaluated using MTT assay. (b) Flow cytometry analysis of apoptotic cell in TFK1 cells. (c) Quantification of apoptotic cells. (d) Detection of caspase-3 activity. (e) Determination of Bax, Bcl-2, and cleaved-Caspase3 protein expression using western blot. *BAX* (f) and *BCL2* (g) mRNA levels in TFK1 cells were detected using qRT-PCR.

### BMF is a direct target of miR-421

**Figure 4.**
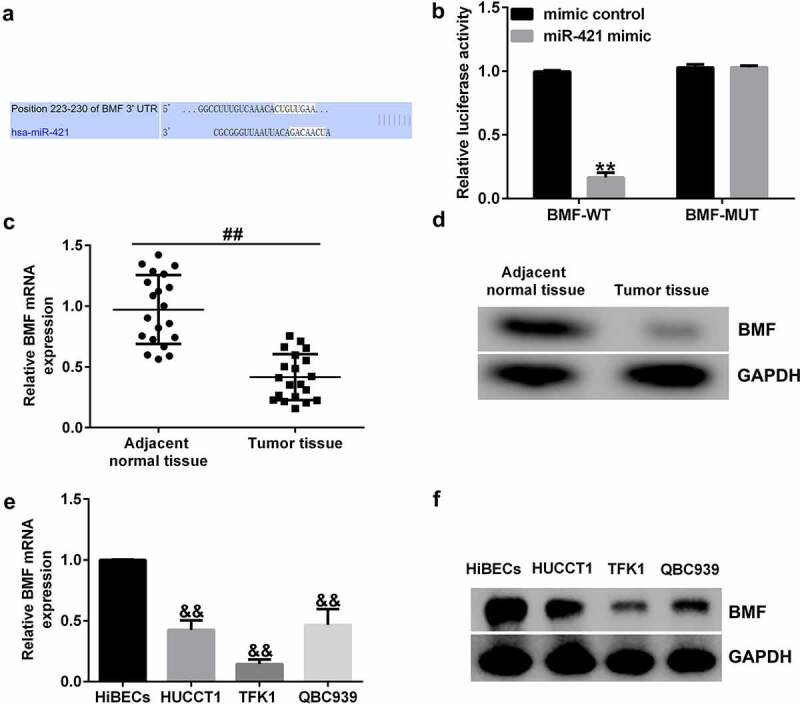
BMF binds to the 3’-UTR of miR-421.

To further identify the role of miR-421 in CCA, we performed a bioinformatics analysis using TargetScan to identify potential targets and found that *BMF* was a latent target of miR-421 (Figure 4(a)). Furthermore, dual-luciferase reporter assay revealed that miR-421 plasmid markedly reduced the luciferase activity of BMF-WT but had no obvious effect on BMF-MUT (Figure 4(b)). In addition, we found that BMF was downregulated in CCA tissues (Figure 4(c) and (d)) and cell lines (HUCCT1, TFK1 and QBC939) compared with HiBECs (Figure 4(e) and (f)). Our findings indicate that *BMF* is a direct target of miR-421.

(a) Schematic of BMF binding site at the miR-421 3’-UTR. (b) Dual-luciferase reporter gene system confirmed that BMF was a direct target of miR-421. (c and e) qRT-PCR analysis of *BMF* level in CCA tissues and cell lines. (d and f) Western blot analysis of BMF expression in CCA tissues and cell lines.

### BMF-siRNA reverses the effects of miR-421 inhibitor on BMF expression

**Figure 5.**
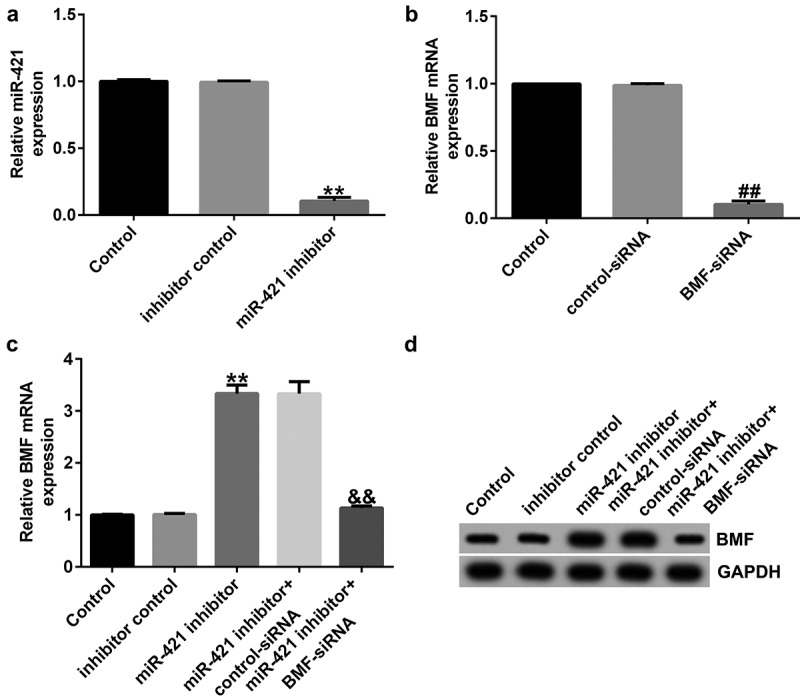
miR-421 negatively regulates BMF levels in TFK1 cells.

Having explored the relationship between *BMF* and miR-421, we then explored the underlying mechanisms of BMF in TFK1 cells. Inhibitor control, miR-421 inhibitor, BMF-siRNA, or control-siRNA was transfected into TFK1 cells for 24 h. qRT-PCR analysis showed that miR-421 inhibitor inhibited miR-421 levels in TFK1 cells (Figure 5(a)). Moreover, BMF-siRNA markedly decreased BMF mRNA levels and protein expression (Figure 5(b)) as compared to control-siRNA. Results from qRT-PCR and western blot suggested that miR-421 inhibitor significantly enhanced the mRNA levels and protein expression of BMF, which was abolished by BMF-siRNA transfection (Figure 5(c) and (d)). Taken together, the results indicate that BMF-siRNA reversed the effects of miR-421 inhibitor on *BMF* expression.

Inhibitor control, miR-421 inhibitor, BMF-siRNA, or control-siRNA was transfected into TFK1 cells for 24 h. (a) Effect of miR-421 expression on mRNA levels was evaluated in TFK1 cells transfected with inhibitor control or miR-421 inhibitor. (b) Effect of BMF expression on mRNA levels was determined in BMF-siRNA or control-siRNA transfected TFK1 cells. (c and d) qRT-PCR and western blot analysis of BMF in TFK1 cells transfected with miR-421 inhibitor or miR-421 inhibitor+BMF-siRNA.

### miR-421 inhibitor suppresses proliferation and induces apoptosis in TFK1 cells through BMF

**Figure 6.**
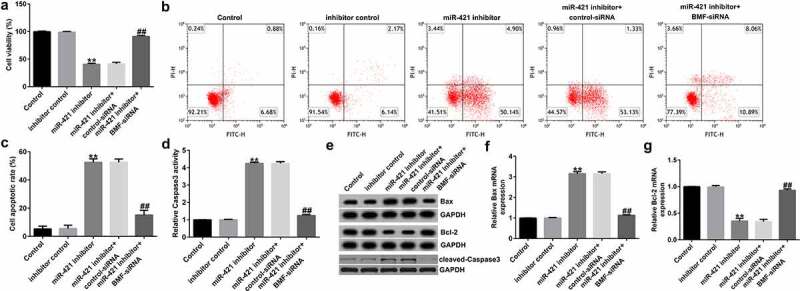
miR-421 downregulation inhibits proliferation and promotes apoptosis in TFK1 cells via BMF.

To further reveal the underlying mechanism of miR-421 in CCA, we assessed the roles of BMF on TFK1 cell growth and apoptosis. TFK1 cells were transfected with inhibitor control, miR-421 inhibitor, BMF-siRNA, or control-siRNA for 24 h. Results from MTT and flow cytometry analysis demonstrated that the miR-421 inhibitor significantly inhibited cell viability (Figure 6(a)) and induced apoptosis (Figure 6(b) and (c)) compared to the inhibitor control. Meanwhile, miR-421 inhibitor enhanced the activity of caspase-3 (Figure 6(d)), promoted Bax and cleaved-Caspase3 expression (Figure 6(e) and (f)), and reduced Bcl-2 levels in TFK1 cells (Figure 6(e)–(g)) compared to the inhibitor control, and these findings were reversed by BMF-siRNA. Our findings suggest that circSETD3 suppresses proliferation and induces apoptosis in TFK1 cells through the miR-421/BMF axis.

TFK1 cells were transfected with inhibitor control, miR-421 inhibitor, BMF-siRNA or control-siRNA for 24 h. (a) Cell proliferation was assessed by MTT assay. (b) Apoptotic cells were evaluated using flow cytometry analysis. (c) Quantification of apoptotic cells. (d) Detection of caspase-3 activity. (e) Western blot analysis of Bax, Bcl-2, and cleaved-Caspase3 expression in TFK1 cells. mRNA levels of *BAX* (f) and *BCL2* (G) were determined using qRT-PCR.

## Discussion

CCA, also known as bile duct cancer, originates from bile duct epithelial cells and is second to hepatocellular carcinoma in terms of incidence. There are approximately 0.6 million newly confirmed cases, and nearly 400,000 patients die of liver cancer annually [24]. In recent years, many therapeutic technologies, such as surgery [25], chemotherapy [26], and therapeutic liver transplantation [27], have been identified as major viable methods for CCA treatment. However, patients with CCA generally have a poor prognosis, and the five-year survival rate remains very low [5]. Although multiple biomarkers have been identified for CCA treatment, availability of prognostic factors and effective treatment is still lacking. Therefore, novel prognostic markers or effective therapies that can provide more clinical evidence for CCA treatment are urgently needed.

Moreover, the prognostic values of various molecules including gene, miRNAs, and circRNAs were reported to be involved in CCA [28]. CircRNAs, a novel type of RNA, have a covalent closed-loop structure that plays important roles in many tumors, including CCA. Recently, Xu et al. indicated that circ_ASPH promotes CCA growth and metastasis via the miR-581/ATP-binding cassette transporter G1 signaling pathway [29]. It was also found that downregulation of circular RNA hsa_circ_0001649 regulates CCA cell proliferation, migration, and invasion [30]. However, whether circSETD3 is associated with the progression of CCA remains poorly understood. In addition, increasing studies have verified that circRNAs exert functions by interacting with miRNAs as a sponge in tumor, including CCA. A number of investigations have confirmed that miR-421 plays vital roles in the progression of human cancers, and many genes have been identified as targets of miR-421. CircSETD3 acts as a miR-421 sponge to inhibit hepatocellular carcinoma growth [11], but it is still unknown whether circSETD3 acts as an important regulator in CCA by targeting miR-421.

In this study, we first evaluated the expression of circSETD3 and miR-421 in 20 pairs of CCA tissues and adjacent nontumor tissues as well as CCA cell lines and HiBECs by qRT-PCR assay. The results indicated that circSETD3 was downregulated while miR-421 was upregulated in CCA tissues and cell lines compared with the control group. These findings are consistent with those of other reports, suggesting that circSETD3 acts as a tumor inhibitor in cancer. For example, Tian et al. found that circSETD3 hindered bladder cancer cell growth, migration, and stem cell properties by targeting miR-641 [31]. Thus, we inferred that circSETD3 upregulation may prevent tumorigenesis. CircSETD3-plasmid, control-plasmid, miR-421 mimic, or mimic control were transfected into TFK1 cells for 24 h. Our data revealed that circSETD3-plasmid promoted circSETD3 levels and reduced miR-421 expression, and this inhibition was reversed by transfection with miR-421 mimic.

In recent years, various reports have confirmed that circRNAs are involved in regulating cell differentiation, proliferation, and apoptosis and are identified as new biomarkers in tumors [32,33]. Functional assays suggested that overexpression of circSETD3 remarkably suppressed proliferation and induced apoptosis in TFK1 cells, proving its tumor-inhibiting effect. Detection of caspase-3 activity demonstrated that it was conspicuously enhanced. Bax and Bcl-2 are important regulators of apoptosis and ultimately lead to apoptotic cell death [34]. This is in accordance with our data that circSETD3-plasmid obviously decreased Bcl-2 expression and promoted Bax and cleaved-Caspase3 expression. However, these effects on TFK1 cells were reversed after transfection with miR-421 mimic. These findings demonstrate that circSETD3 inhibits the activity of CCA cells and induces apoptosis by regulating miR-421.

MiRNAs often exert their functions by interacting with their target genes [35,36]. To further investigate the mechanism of miR-421 in CCA, we predicted its potential targets and found that BMF directly interacted with miR-421. We also found that BMF levels in CCA tissues and TFK1 cells were lower than those in paracarcinoma tissues and HiBECs. To elucidate the relationship between *BMF* and miR-421 in CCA cells, TFK1 cells were transfected with inhibitor control, miR-421 inhibitor, BMF-siRNA, or control-siRNA for 24 h. We observed that miR-421 inhibitor inhibited miR-421 levels in TFK1 cells, while BMF-siRNA markedly decreased BMF mRNA levels and protein expression compared with control-siRNA. Moreover, the results of qRT-PCR and western blot demonstrated that miR-421 inhibitor significantly enhanced the mRNA levels and protein expression of BMF, and this increase was abolished by BMF-siRNA. We also observed that miR-421 inhibitor remarkably suppressed proliferation and induced apoptosis in TFK1 cells, increased caspase-3 activity, decreased Bcl-2 expression, and promoted Bax and cleaved-Caspase3 expression, and these effects were reversed by BMF-siRNA. From these results, we conclude that inhibition of miR-421 inhibits proliferation and induces apoptosis in CCA cells by promoting BMF expression.

There were also some limitations of this study. For example, the correlation between circSETD3 and miR-421were not present in this study. Besides, cell viability was only detected using MTT assay, and cell proliferation detected by colony formation and EdU assays will make our results more convincing. Moreover, we only investigated the role of circSETD3 and miR-421 in only one CCA cell line (TFK1 cells), and *in vivo* experiments were not performed. We will further explore the role of circSETD3 and miR-421 in other CCA cell lines and CCA animal model in our next study.

## Conclusion

Our data show for the first time that circSETD3 is downregulated and miR-421 is upregulated in CCA cells and tumor tissues, and circSETD3 inhibits proliferation and induces apoptosis in CCA cells via regulation of the miR-421/BMF axis. This study elucidates the pathogenesis of CCA and may provide an innovative insight for CCA treatment.

## Data Availability

The datasets used and/or analyzed during the current study are available from the corresponding author on reasonable request.
